# Supplementary material for: Optical single-shot readout of spin qubits in silicon
Source: Nat Commun. 2025 Jan 2;16:64. doi: 10.1038/s41467-024-55552-9 (PMC11695859; doi:10.1038/s41467-024-55552-9)
Supplement: Supplementary file 1 — Supplementary Information [file 41467_2024_55552_MOESM1_ESM.pdf]

Supplementary Information: Optical single-shot readout of spin qubits in silicon

Andreas Gritsch, Alexander Ulanowski, Jakob Pforr, and Andreas Reiserer*
Max-Planck-Institute of Quantum Optics, Hans-Kopfermann-Straße 1, 85748 Garching, Germany and
TUM School of Natural Sciences, Department of Physics and Munich Center for Quantum Science and Technology (MCQST),
Technical University of Munich, James-Franck-Straße 1, 85748 Garching, Germany

Supplementary Note. 1. INITIAL DEVICE CHARACTERIZATION

For an initial characterization of the device, we use Argon gas condensation to tune the cavity to 194 954.05(10) GHz — on resonance with the center of the inhomogeneous distribution of the erbium transitions of site A [1] in the absence of a magnetic field. After pulsed, resonant excitation [2] using the setup described above, we record the fluorescence spectrum (shown in Fig. 1b), in which we observe several peaks that originate from individual dopants, confirmed by autocorrelation function measurements that show clear antibunching, $g^{(2)}(0) < 0.5$, on the isolated peaks. The emitters differ in frequency because of their different local strain environments. The observed inhomogeneous distribution of ≈ 1 GHz is consistent with previous measurements with ensembles [1]. It is narrower than the cavity linewidth full-width-at-half-maximum (FWHM), such that the emission of all dopants in the resonator is enhanced in this measurement. From the observed number of emitters, the resonator mode volume and the implanted dose, we find that less than 1 % of the emitters are integrated in site A, consistent with our earlier work [1].

Supplementary Note. 2. CHOICE OF THE DOPANT

As shown in Fig. 1b, three erbium emitters in the studied device are spectrally isolated from the others and can thus be individually addressed [3, 4]: the most red-detuned dopant (in the following: red), the most blue-detuned dopant (blue) and the dopant at a detuning of about 0.12 GHz (center). We find that these three isolated emitters have Lorentzian spectral-diffusion linewidths of 47(1) MHz (red), 33(2) MHz (center) and 13.5(5) MHz (blue) FWHM. The cavity is tuned on resonance with each of these emitters using the temperature tuning scheme (see Methods) for additional characterization measurements. We find that the red dopant exhibits the best single-photon purity, $g^{(2)}(0) = 0.019(1)$, while the others have slightly worse values of 0.07(1) and 0.09(1) for the center and blue emitter, respectively. Furthermore, the red dopant shows the strongest Purcell enhancement, $P = 177(2)$, compared to values of 89(2) (center) and 29(1) (blue). The observed variation of the Purcell enhancement between the different emitters is expected because of their integration at random positions within the cavity mode [2]. As a result, the ratio between the lifetime-limited linewidth and the spectral diffusion linewidth is superior for the red dopant. Combining all aspects, the red dopant exhibits the most promising properties in this device and is thus used throughout this work.

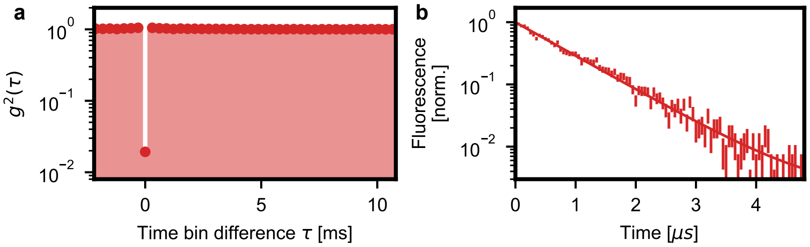

Supplementary Fig. 1. **Characterization of the most red-detuned dopant in Fig. 1. a,** Autocorrelation measurement. Without an external magnetic field and by applying the same methods detailed in [2], we find an anti-bunching at zero delay of $g^{(2)}(0) = 0.019(1)$. **b,** Optical lifetime. The time-resolved fluorescence is recorded after resonant excitation and then fitted with a single exponential. From this fit, we extract an optical excited state lifetime of 0.803(11) µs. The emitter studied in this work thus exhibits the shortest optical lifetime that could be demonstrated for any erbium dopant. Error bars: 1 SD.

* andreas.reiserer@tum.de

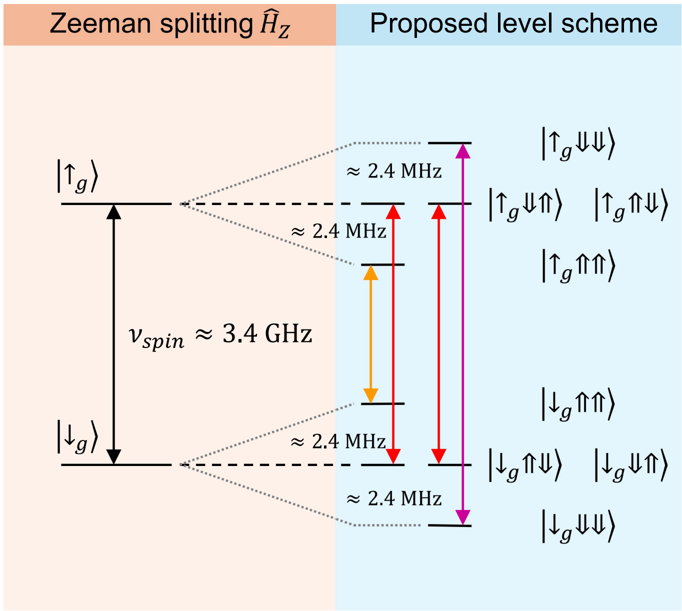

Supplementary Fig. 2. **Proposed level scheme with two coupled nuclear spins.** A three-fold splitting of the spin transition is observed in Figure 3a, where the center transition has a higher amplitude. A possible explanation is that the electronic spin interacts with two ^{29}Si nuclear spins that are in close proximity and exhibit a similar parallel hyperfine coupling of ~ 1 MHz. Assuming dipolar interactions, the observed splitting is compatible with ^{29}Si positions in the first and second shell around the erbium dopant. A detailed investigation is left for future work.

[1] A. Gritsch, L. Weiss, J. Früh, S. Rinner, and A. Reiserer, Phys. Rev. X **12**, 041009 (2022).
[2] A. Gritsch, A. Ulanowski, and A. Reiserer, Optica **10**, 783 (2023).
[3] S. Chen, M. Raha, C. M. Phenicie, S. Ourari, and J. D. Thompson, Science **370**, 592 (2020).
[4] A. Ulanowski, B. Merkel, and A. Reiserer, Sci. Adv. **8**, eabo4538 (2022).
